# Assessing dengue fever risk in Costa Rica by using climate variables and machine learning techniques

**DOI:** 10.1371/journal.pntd.0011047

**Published:** 2023-01-13

**Authors:** Luis A. Barboza, Shu-Wei Chou-Chen, Paola Vásquez, Yury E. García, Juan G. Calvo, Hugo G. Hidalgo, Fabio Sanchez

**Affiliations:** 1 Centro de Investigación en Matemática Pura y Aplicada - Escuela de Matemática, Universidad de Costa Rica, San José, Costa Rica; 2 Centro de Investigación en Matemática Pura y Aplicada - Escuela de Estadística, Universidad de Costa Rica, San José, Costa Rica; 3 Centro de Investigación en Matemática Pura y Aplicada, Universidad de Costa Rica, San José, Costa Rica; 4 Department of Public Health Sciences, University of California Davis, California, United States of America; 5 Centro de Investigaciones Geofísicas and Escuela de Física, Universidad de Costa Rica, San José, Costa Rica; National Center for Atmospheric Research, UNITED STATES

## Abstract

Dengue fever is a vector-borne disease affecting millions yearly, mostly in tropical and subtropical countries. Driven mainly by social and environmental factors, dengue incidence and geographical expansion have increased in recent decades. Therefore, understanding how climate variables drive dengue outbreaks is challenging and a problem of interest for decision-makers that could aid in improving surveillance and resource allocation. Here, we explore the effect of climate variables on relative dengue risk in 32 cantons of interest for public health authorities in Costa Rica. Relative dengue risk is forecast using a Generalized Additive Model for location, scale, and shape and a Random Forest approach. Models use a training period from 2000 to 2020 and predicted climatic variables obtained with a vector auto-regressive model. Results show reliable projections, and climate variables predictions allow for a prospective instead of a retrospective study.

## Author summary

Dengue fever is a vector-borne viral disease endemic to tropical and subtropical countries. The virus is transmitted by female *Aedes* mosquitoes and affects approximately 100 million people every year. Although most infections are mild or asymptomatic, some may cause severe symptoms, leading to a higher risk of death. In the affected countries, the challenges associated with preventing and controlling dengue outbreaks have highlighted the need for novel tools. In this context, using statistical tools with climate and epidemiological information makes it possible to provide timely information to public health officials about the risk of dengue outbreaks, allowing the optimization of resources and preventive and non-reactive decision-making.

## Introduction

Dengue virus transmission represents a public health challenge for countries in tropical and subtropical regions worldwide [1]. For the past decades, the increasing geographical spread of the pathogen and its two main vectors, *Aedes aegypti* and *Aedes albopictus* [2], has led to the development and implementation of multiple prevention and control measures [3]. However, it is difficult to achieve timely, effective, and sustainable strategies due to the complex interactions and constant variations in population mobility and socioeconomic, demographic, environmental, and climate factors that modulate the spatial and temporal distribution of the disease.

Researchers worldwide are increasingly working towards developing innovative, tailored, and cost-effective tools that enhance the design of public health policies for vector-borne diseases founded upon the rapid systematization and analysis of information, as well as an increase in interdisciplinary collaboration [4]. In these efforts, statistical and machine learning techniques are increasingly used for public health surveillance and epidemiological modeling [5]. Through computational algorithms, this branch of artificial intelligence facilitates integrating scientific knowledge, processing large databases, learning from past documented reported cases, and ultimately projecting transmission tendencies to identify and target the most vulnerable at-risk areas. Dengue is a climate-sensitive disease where changes in temperature, humidity, and precipitation affect the mosquito’s biology, behavior, and availability to reproduce, develop, propagate the virus, and interact with the human host [6–8]. Using satellite imagery and weather monitoring as input data in machine learning models and other statistical learning approaches has shown promising results [9, 10] that could effectively predict the relative risk of dengue transmission.

Costa Rica is a country of 5,163,021 inhabitants [11], administratively divided into seven provinces and 83 cantons, of which 32 cantons are of interest to health authorities due to the high dengue incidence. The various micro-climates in Costa Rica provide ideal conditions for the mosquito vector to thrive. They are making it necessary to customize dengue transmission risk analysis to improve prevention and control measures implemented by health authorities.

In this article, we show the results of using two different statistical modeling approaches, the Generalized Additive Model, for location, scale, and shape (GAMLSS) and Random Forest (RF), to forecast the relative risk of dengue infections in 32 cantons of Costa Rica. The analysis is a continuation of previous work in [12], where an initial approach using Generalized Additive Models (GAM) and RF allowed us to retrospectively predict the relative risk of dengue for 2017 in five diverse climate cantons, using as input the information of five weather stations provided by the National Meteorological Institute [12].

## Data description

### Dengue cases

Data of clinically suspected and confirmed monthly cases of dengue fever in Costa Rica is collected from all the local country’s administrative areas (cantons), covering the years 2000–2021, and provided by the Ministry of Health [13]. To quantify the relative incidence of dengue cases at the *i*-th canton compared with the incidence in the country at time *t* (monthly basis), we use the relative risk (*RR*):

$$\begin{array}{c} RR_{i,t}=\frac{\frac{{\text{Cases}}_{i,t}}{{\text{Population}}_{i,t}}}{\frac{{\text{Cases}}_{CR,t}}{{\text{Population}}_{CR,t}}}, \end{array}$$

where Cases_*i*,*t*_ (Population_*i*,*t*_) and Cases_*CR*,*t*_ (Population_*CR*,*t*_) are the number of observed dengue cases (population size) at canton *i* and country-level respectively, at time *t*. We use the relative risk instead of the attack rate to compare the dengue incidence among cantons relative to the incidence observed in the whole country.

**Fig 1:**
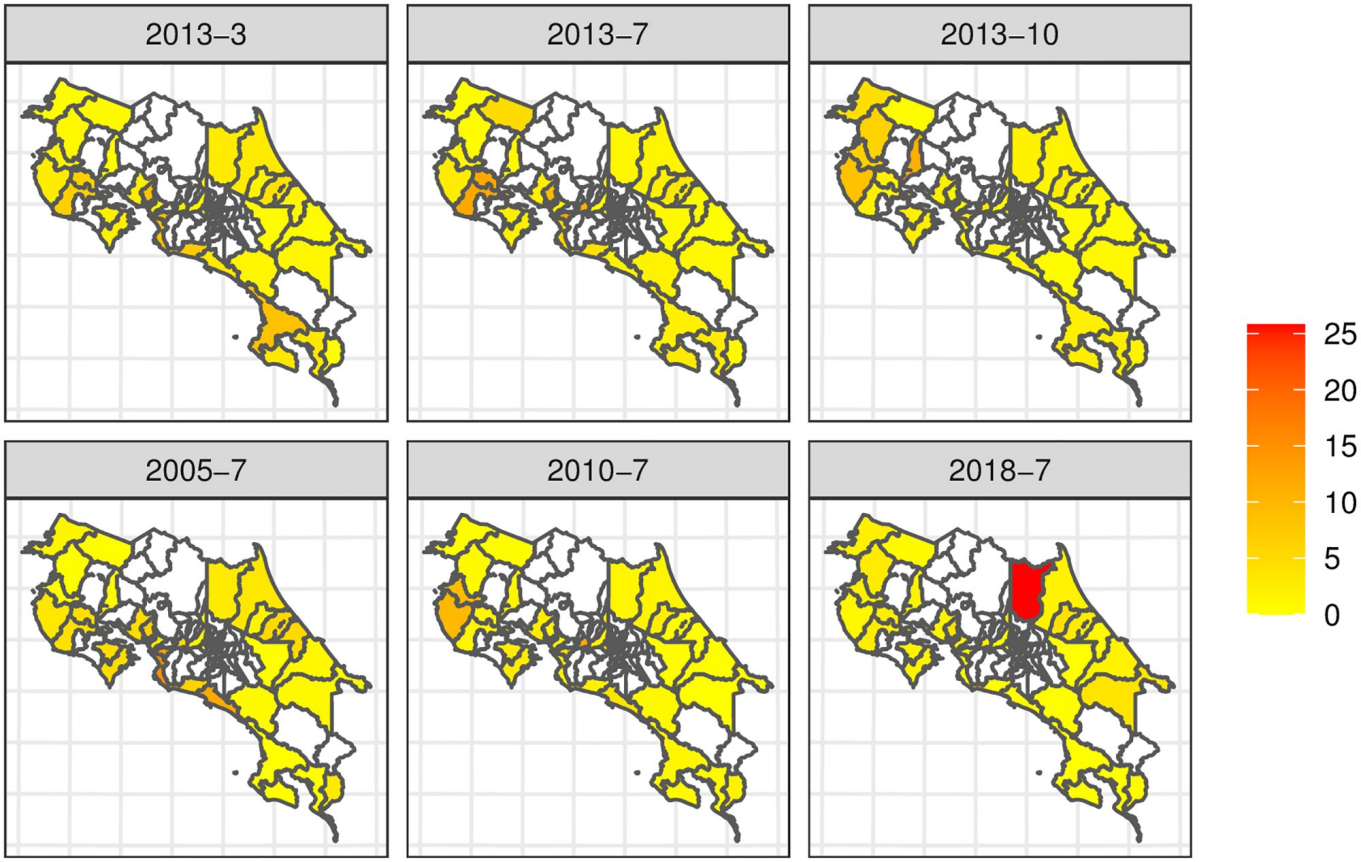
The Relative Risk (*RR*) over the 32 cantons in the study for different months and years of available data. We show three months for 2013 (top panels) and July for three different years (bottom panels). The map was created using R software (shapefile found here: https://hub.arcgis.com/datasets/741bdd9fa2ca4d8fbf1c7fe945f8c916_0/explore). The license is public (https://hub.arcgis.com/datasets/geotec::distritos-de-costa-rica/about).

The overall behavior of the relative risks, at three specific months in 2013 (first row), and three specific years in July (second row) is shown in Fig 1.

### Climate variables

1. Daily Precipitation estimates (*P*_*i*,*t*_) were used to index land surface rainfall. Data were obtained from the Climate Hazards Group InfraRed Precipitation with Station data (CHIRPS); see [14]. Due to the high-resolution spatial nature of this dataset (5km by 5km), we were able to compute monthly cumulative rainfall estimates for each canton by adding the exact estimate over smaller administrative areas (*distritos*).
2. El Niño Southern Oscillation (ENSO, *S*_*i*,*t*_), also known as the SSTA index. Weekly data was obtained from the Climate Prediction Center (CPC) of the United States National Oceanographic and Atmospheric Administration (NOAA) (see [15]).
3. Normalized Difference Vegetation Index (NDVI, *N*_*i*,*t*_), an index of the greenness of vegetation for a 16-day time resolution and 250m spatial resolution. It was obtained from the Moderate Resolution Imaging Spectroradiometer (MODIS) satellite and available through the MODISTools R package (see [16]).
4. Daytime Land Surface Temperature (LST, *L*_*i*,*t*_) in Kelvin degrees for an 8-day time resolution and 1km spatial resolution, obtained using the same resources as the NDVI covariate.
5. Tropical Northern Atlantic Index (TNA, *TN*_*i*,*t*_). Anomaly index of the sea-surface temperature over the eastern tropical North Atlantic Ocean (see [17]). This index is used because previous work in the region, such as [18], suggested that the inclusion of SST information from the Caribbean/Atlantic improves performance compared to forecasts produced with only Pacific Ocean ENSO conditions.

The data that support the findings of this study are publicly available from github with the identifier https://github.com/luisbarboza27/DengueCR_ST_Prediction

## Methods

### Fitting stage

To model the relationship between climate covariates and relative dengue risk in a canton, we incorporate the historical delayed associations between those variables by applying a Distributed Lag Non-Linear Model (DLNM) framework [19, 20]. The DLMN consists of a bi-dimensional space of functions that specifies an exposure-lag-response function *f* ⋅ *w*(*x*, *l*), which depends on the predictor *x* along the time lags *l* in a combined way. This combination specifies a non-linear and delayed association between climate covariate and dengue incidence. For each covariate, we consider a maximum exposure of 18 months in its lag representation, based on the cross-correlation and wavelet behavior among the series (see [21]) and a b-spline or linear basis representation on the variable space. We use the R package dlnm [22] for all calculations.

The model’s structure is as follows:

$$\begin{array}{c} RR_{t}∼f\left(RR_{t-1},C_{1}P_{t},C_{2}S_{t},C_{3}N_{t},C_{4}L_{t},C_{5}TN_{t},M_{t}\right) \end{array}$$ (1)

where *f* is a function depending on the method employed, the matrices *C*_*i*_ are defined in terms of the DLNM representation, and *M*_*t*_ is a factor-type variable describing the monthly fixed effect (the unit of Time *t* is in months). The first method that we use for *f* is the GAMLSS. It represents a generalization of the GAM method used in [12]. It is a flexible class of statistical framework where the location, scale, skewness, and kurtosis parameters from the response variable distribution can be modeled as an additive function of covariates [23]. In this particular case, the model is written as:

$$\begin{array}{cc} RR_{t} & \overset{ind}{∼}D\left(\mu ,\sigma ,\nu \right)\\ g_{1}\left(\mu \right) & =\beta_{10}+\beta_{11}RR_{t-1}+\beta_{12}C_{1}P_{t}+\beta_{13}C_{2}S_{t}+\beta_{14}C_{3}N_{t}+\beta_{15}C_{4}L_{t}+\beta_{16}C_{5}TN_{t}+\beta_{17}M_{t}\\ g_{2}\left(\sigma \right) & =\beta_{20}\\ g_{3}\left(\nu \right) & =\beta_{30} \end{array}$$ (2)

The response variable is distributed as a three-parameter distribution D: the location (*μ*), the scale (*σ*), a parameter related to the skewness of the distribution (*ν*), and link functions (*g*_*i*_ for *i* = 1, 2, 3).

Because the monthly relative risk of dengue is a non-negative skewed variable with a significant frequency of zeros (16.2%), mixed distributions with a positive domain and positive probability at zero are appropriate for modeling purposes. The zero-adjusted gamma distribution (ZAGA) and the zero-adjusted inverse Gaussian (ZAIG) are considered. The results for both choices are similar. Therefore, we only show our results for the ZAGA distribution.

The mixed continuous-discrete probability density defines the ZAGA density function:

$$f_{Y}\left(y\right)=\{\begin{array}{c} \nu \;\;\;\;\;\;\;\;\;\;\;\;\;\;\;\;\text{if}\;y=0\\ \left(1-\nu \right)f_{W}\left(y\right)\;\;\text{if}\;\;0<y<\infty \end{array}$$

for 0 ≤ *y* < ∞, where *W* ∼ *GA*(*μ*, *σ*) is a gamma distribution with 0 < *μ* < ∞, 0 < *σ* < ∞ and 0 < *ν* < 1, i.e.

$$\begin{array}{c} f_{W}\left(y|\mu ,\sigma \right)=\frac{1}{{\left(\sigma^{2}\mu \right)}^{1/\sigma^{2}}}\frac{y^{\frac{1}{\sigma^{2}}-1}e^{-y/(\sigma^{2}\mu )}}{\Gamma (1/\sigma^{2})}, \end{array}$$

for *y* > 0, *μ* > 0 and *σ* > 0. The advantage of this parametrization is that *E*(*W*) = *μ* and *V*(*W*) = *σ*^2^*μ*^2^. For the GAMLSS specification, *ZAGA*(*μ*, *σ*, *ν*) defines the log link functions for *μ* and *σ*, i.e., *g*_1_(*μ*) = log(*μ*) and *g*_2_(*σ*) = log(*σ*); and the logit link function for *ν*, i.e. *g*_3_(*ν*) = log[*ν*/(1 − *ν*)].

The second method uses an RF approach. This method is based on the construction of bootstrapped ensemble of regression trees and combined such that the prediction variance can be reduced (see [24] and [25]). One of the main advantages of this method is the reduced number of tuning parameters that eases its computational manipulation and stability [24].

The fitting process of the GAMLSS and RF models was performed with the R packages gamlss [26], and ranger [27], respectively.

### Prediction stage

**Fig 2:**
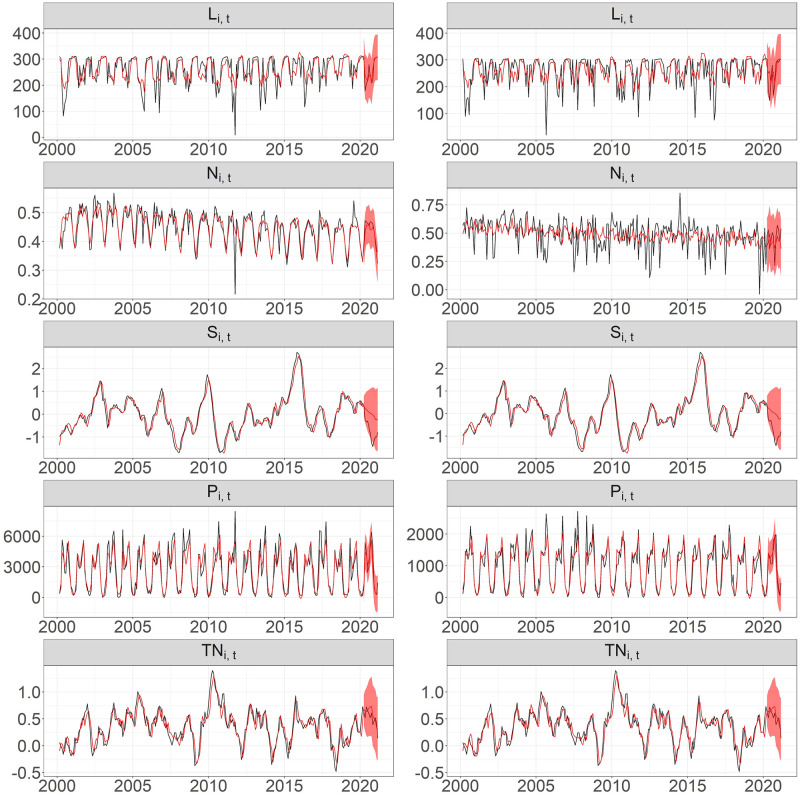
Observed climate covariates and forecast values at two specific cantons: Alajuela (left panels) and Quepos (right panels). Black line: observed climate covariates, red line: forecast values, and red shaded areas: 95% confidence regions.

Once Eq (1) is fitted over a certain calibration period using any of the two methodological alternatives, we forecast the relative risk over a testing period using the past information of climatic covariates and the relative risk itself. Provided that the response variable in (1) depends on the current values of the climatic covariates, it is crucial to select an appropriate method to obtain the climate predictions in the near future that can supply accurate inputs to our predictive model under both methodologies. Since the climate covariates used in this study are highly correlated, a suitable method to describe and predict their interaction is the vector auto-regressive (VAR) model (see more details in [28]). For each canton, we include the trend and seasonal factors to fit a VAR model and select the best lag order based on the BIC criterion over the training period (which is the same period as the one used for the fitting (1)). Then, we jointly forecast over the testing period the covariates. In Fig 2, we illustrate the observed climate covariates and their forecast values at Alajuela and Quepos. Together with the predicted relative risks, these predictions provide forecasts of the dependent variable over the testing period. Finally, to assess the prediction uncertainty, we apply a non-parametric bootstrap [29] and construct prediction intervals using the corresponding forecasts for each bootstrap step without considering the uncertainty due to the covariate prediction.

### Model comparison

We use two different metrics to compare the predictive performance of each methodology for each fixed location. The normalized Mean-Squared Error (*NRMSE*):

$$\begin{array}{c} NRMSE=\sqrt{\frac{1}{m\bar{RR}}\sum_{t=1}^{m}{\left(RR_{t}-{\hat{RR}}_{t}\right)}^{2}}, \end{array}$$

where *m* is the number of months in the testing period, $\bar{RR}$ is the mean relative risk over the same period, and $\hat{RR}$ is the estimated relative risk according to any of the two models. The normalized Interval Score at *α* level (*NIS*_*α*_) is the normalized version of the Interval Score (see [30] and [31]). While *NRMSE* compares the precision between point forecast and the observed relative risk, *NIS*_*α*_ is a metric that compares the upper and lower limits of a prediction interval associated with (1 − *α*)% confidence against the observed relative risk. Therefore, we can compare different locations regardless of the scale of their corresponding relative risk:

$$\begin{array}{c} NIS_{\alpha }=\frac{1}{m\bar{RR}}\sum_{t=1}^{m}[\left(U_{t}-L_{t}\right)+\frac{2}{1-\alpha }\left(L_{t}-RR_{t}\right)\cdot 1_{RR_{t}<L_{t}}+\frac{2}{1-\alpha }\left(RR_{t}-U_{t}\right)\cdot 1_{RR_{t}>U_{t}}], \end{array}$$

where *U*_*t*_ and *L*_*t*_ are the upper and lower limits of the prediction interval, respectively. The latter metric is more complete than the former in evaluating the models’ predictive capacity when the uncertainty is summarized through a predictive interval [31].

## Results

We used the dengue and climate data described above to fit the model in (1) using both the GAMLSS and RF methodologies. The training period includes monthly observations for the 32 cantons in the study from January 2000 to December 2020. This period was considered due to available satellite data and epidemiological information. The DLNM basis was chosen to be linear in the variable and lag space for the TNA index, LST, and NDVI, whereas a B-spline basis is assumed for the variable space, which is linear for the lag space for precipitation and ENSO. These choices allow an acceptable balance between the complexity of the models and predictive precision over all the locations.

Once the transformed covariates in (1) are determined, we fitted both methodologies over the 32 locations individually and adjusted a VAR model using the climate information for each location over the training period to obtain predicted values of the covariates. We then predicted the relative risk of dengue for the first three months of 2021. Fig 2 shows the fitted values and predictions of the climate covariates for Alajuela and Quepos. We observed that features like trend and seasonality of the multiple time series are well-captured for the testing period for all the cantons.

**Fig 3:**
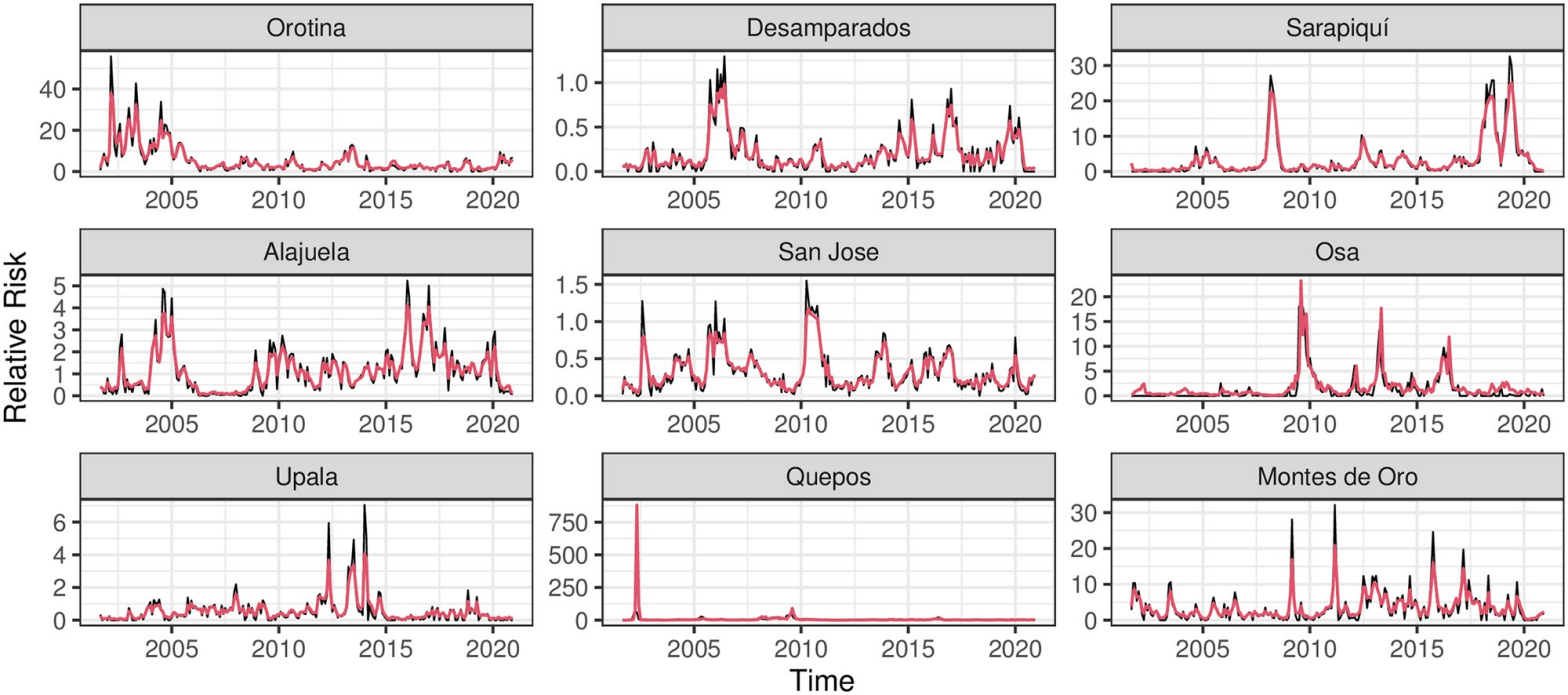
Comparison over the fitting period. Upper six panels: best cantons according to NIS metric. Lower three panels: worst cantons according to NIS metric. Black line: observed *RR*, red line: estimated *RR* and red shaded area: 95%-confidence predictive region.

**Fig 4:**
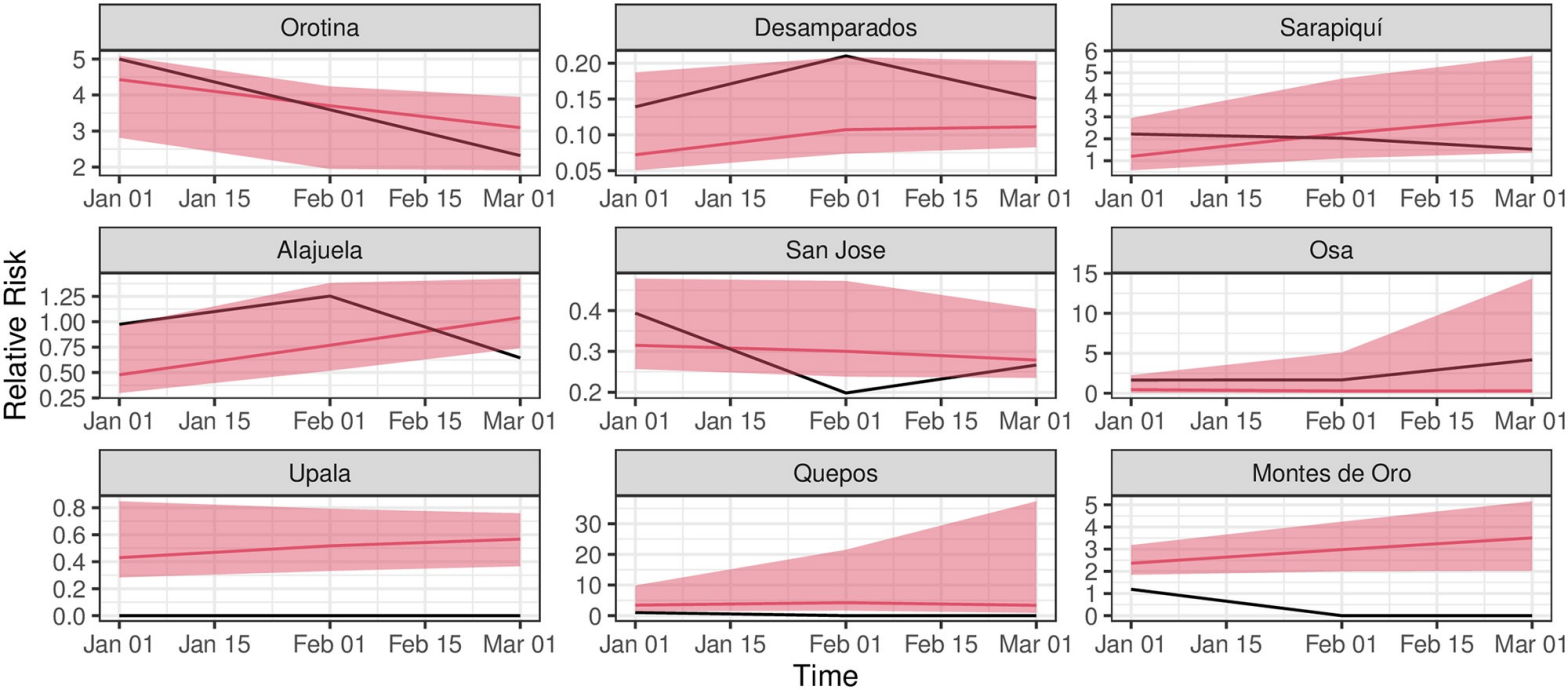
Forecast comparison over the testing period (2021). Upper six panels: best cantons according to NIS metric. Lower three panels: worst cantons according to NIS metric. Black line: observed *RR*, red line: estimated *RR* and red shaded area: 95%-confidence predictive region.

Due to the auto-regressive nature of the model in (1), the predicted value of *RR*_*t*_ as a covariate was used in the prediction of *RR*_*t*+1_. Once the predicted relative risks over the first three months of 2021 are computed, we compared the observed and predicted values with the *NRMSE* and *NIS*_.95_ metrics and show the behavior of the best six cantons and worst three cantons according to the latter metric, regardless of the method employed. The comparison is shown for the training period in Fig 3 and the testing period in Fig 4. Moreover, there is no significant difference among the methods in the *NIS*_95_ metric: see S1 Fig. We show in S1 Table which model is chosen for each location and their respective metrics, where in general, there is not a model that is predominant over all the locations. Note that there can be differences in the predictive capacity of the climatic covariates for each canton, particularly with the ones that give larger values of the *MSE* and *NIS* metrics.

Together with the expected values of the relative risks in Fig 4, we computed predicted uncertainties at 95%-level using a blocked non-parametric bootstrap [29] with 100 replicates and a block size of six months, over the testing period only.

The fitting performs well in the training period, except for some extreme observations that the model does not capture closely, for example, in Quepos and Montes de Oro. Moreover, the monthly trend over the testing period is well captured in Orotina and Desamparados and partially in Alajuela and San Jose, where for these last two cases, it is captured in two out of three testing points. In the case of Osa, the trend is captured, and the metrics are relatively low. Still, the excess uncertainty at the end of the testing period can be due to the observed behavior during the training period, where this canton suffered localized outbreaks, and the most recent data shows a marked decrease in the relative risk. The model has difficulties fitting both episodes. The uncertainty contains the trend information while it covers most of the observed values through the best-fit cantons.

We also evaluated the capacity of the model to predict high-risk cantons vs. low-risk ones. Using the normalized Kendall distance, we computed the distance among rankings of observed relative risks and expected relative risks obtained by the best individual models of each canton. This exercise was computed with the three-time points of the testing period. In summary, the Kendall distances are respectively 28%, 39%, and 42% for those time points, showing that the ability to classify the model increases with the time horizon and assures that less than half of the cantons are classified accordingly to the observed rankings. However, we model and predict the relative risk for each canton separately, and we did not consider the spatial correlation among the cantons.

Note that we obtained the above results by comparing two modeling alternatives based on the study of [12], which is the first predictive statistical study of dengue data and climatic information in because but we were not able to compare with other alternatives because the study of Vásquez et al. [12], 2020 is the first one of its type (predictive study) using dengue data in Costa Rica.

## Discussion

In this work, we implemented two statistical models, GAMLSS and RF, to predict relative dengue risk in 32 different cantons of interest for public health authorities in Costa Rica, incorporating predictions of climate variables. This approach overcame some limitations of the methodology implemented by Vasquez et al. [12], which is the first predictive study of its type carried out in the country using dengue data. In this new approach, the GAMLSS flexibility allowed capturing the dynamic of relative risks in cantons with low cases and positively skewed. The DLNM framework incorporated the climatic effect using 18 prior months to train the model instead of using a single most significant lag (according to the cross-correlation) of the climatic variables. Furthermore, one of the achievements of predicting climatic variables using a vector auto-regressive (VAR) model is the possibility of performing perspective instead of a retrospective analysis while capturing general features like trend and seasonality on each predicted multiple time series.

In Costa Rica, the dynamics of dengue change geographically and temporally, so it has been necessary to carry out more localized studies to optimize health outcomes and address the specific local conditions that ultimately result in high-risk levels. By training the models with data from 2000 to 2020, our results showed that GAMLSS and the RF models successfully predict relative dengue risk in the testing period (first three months of 2021) in most of the cantons, capturing the trend and seasonality of the multiple time series. Although the model showed good performance in most of the cantons, the model’s predictive capacity had limitations in some cantons, including Montes de Oro, Quepos, and Upala. This cantonal-level analysis highlights the spatial heterogeneity of the effect of climate factors on dengue incidence, which reveals that the effect of those variables on dengue transmission on a local scale might differ from global expectations. The importance of climatic information regarding the incidence of dengue fever has been well established [6–8]. However, a complex interaction of biological, socioeconomic, and environmental factors also impacts dengue transmission [32], creating a substantial spatiotemporal heterogeneity in dengue outbreak intensity. Future studies should consider the incorporation of other no-climate variables such as socioeconomic and ecological factors [33] to improve models’ predictive capacity in regions where climate variables are not enough.

As climate change progresses, extreme weather events such as heatwaves and unusually high rainfall are predicted to be more intense and frequent. A recent study [34] suggests that extreme weather events, including heatwaves, extremely high rainfall, and extremely high relative humidity, may increase the risk of dengue outbreaks. However, more studies about the associations between extreme weather events and outbreaks of vector-borne diseases are needed to understand the correlation. S2, S3 and S4 Figs show a timeline of extreme events registered in Costa Rica from 2011 to 2020, the time series of dengue cases in the 32 cantons considered in this study (S2 Fig), dengue cases of cantons located in the Pacific (S4 Fig), and dengue cases in cantons located in the Atlantic region (S3 Fig). The extreme events registered in 2011, 2012, 2016, and 2017 coincide with high peaks of dengue cases, mainly in the Atlantic cantons. A rigorous analysis is necessary to establish any correlation between those events and dengue outbreaks. But considering extreme weather events may also help develop an effective early warning system for dengue outbreaks, especially in global warming.

The development of reliable early warning systems for dengue epidemics would allow for lowering the economic impact of the disease [35, 36] and better evaluation of the outcomes of prevention programs by the community. The cost of preventive measures has been reported to be less than that of treating an outbreak [36]. Therefore, early prediction tools are valuable because they allow for taking preventative measures and directing and optimizing resources, particularly in countries like Costa Rica, where economic and human resources are limited. A person with mild symptoms of dengue can be disabled on average for seven days [37], reducing the workforce and affecting the income of affected patients. The costs of the Costa Rican Social Security Fund in care for users with dengue were estimated at $20.3 million for 2013, including care for hospitalized patients, medical consultations, and disabilities. The Ministry of Health estimatedat $6.5 million the investment in preventive campaigns and combat actions in the same period [38]. The possibility to forecast an increase in risk can also be used to develop more targeted community-based strategies. Even though community participation has been part of vector control programs since their inception, it has been a challenge to achieve adequate commitment from the population [39]. Therefore, early involvement of different sectors and sharing information on model results with selected communities can potentially allow a more communicative and inclusive approach, generating a greater interest from the community to implement the vector control strategies widely recommended by health officials throughout the country.

Early warning systems (EWS) for vector-borne diseases are incredibly complex due to numerous factors originating from individuals, the environment, the vector, and the disease itself. However, creating reliable forecasting models may lead to fast decision-making processes that trigger disease intervention strategies to minimize the impact on a specific population [40]. A finer study scale with local predictive outbreak risks is necessary because global models may depict the general situation. But, they do not have the necessary detail to drive control strategies at the country scale. Models should include local and historical data and consider local processes that might work differently among regions. This study highlights the potential of GAMLSS and RF for local dengue prediction using climate co-variables but also reveals that these variables, though useful to estimate annual transmission risk, do not fully describe the distribution of dengue occurrence at the country scale. Our model did not consider other local factors such as population counts, income inequality, education, entomological, medical surveillance, and control measures that may be significant for further explaining the spatial distribution of cases.

## Supporting information

S1 Fig Comparison of the distribution of NIS metric among methods. (TIF)

S2 Fig Total dengue cases reported in the country and the timeline of the main extreme events. (TIF)

S3 Fig Total dengue cases reported in the country’s cantons in the Atlantic region and the timeline of the extreme events. (TIF)

S4 Fig Total dengue cases reported in the country’s cantons in the Atlantic region and the timeline of the extreme events. (TIF)

S1 Table Best model for each canton. (XLSX)
