# Supplementary figures and images for: Assessing dengue fever risk in Costa Rica by using climate variables and machine learning techniques
Source: PLoS Negl Trop Dis. 2023 Jan 13;17(1):e0011047. doi: 10.1371/journal.pntd.0011047 (PMC9879398; doi:10.1371/journal.pntd.0011047)

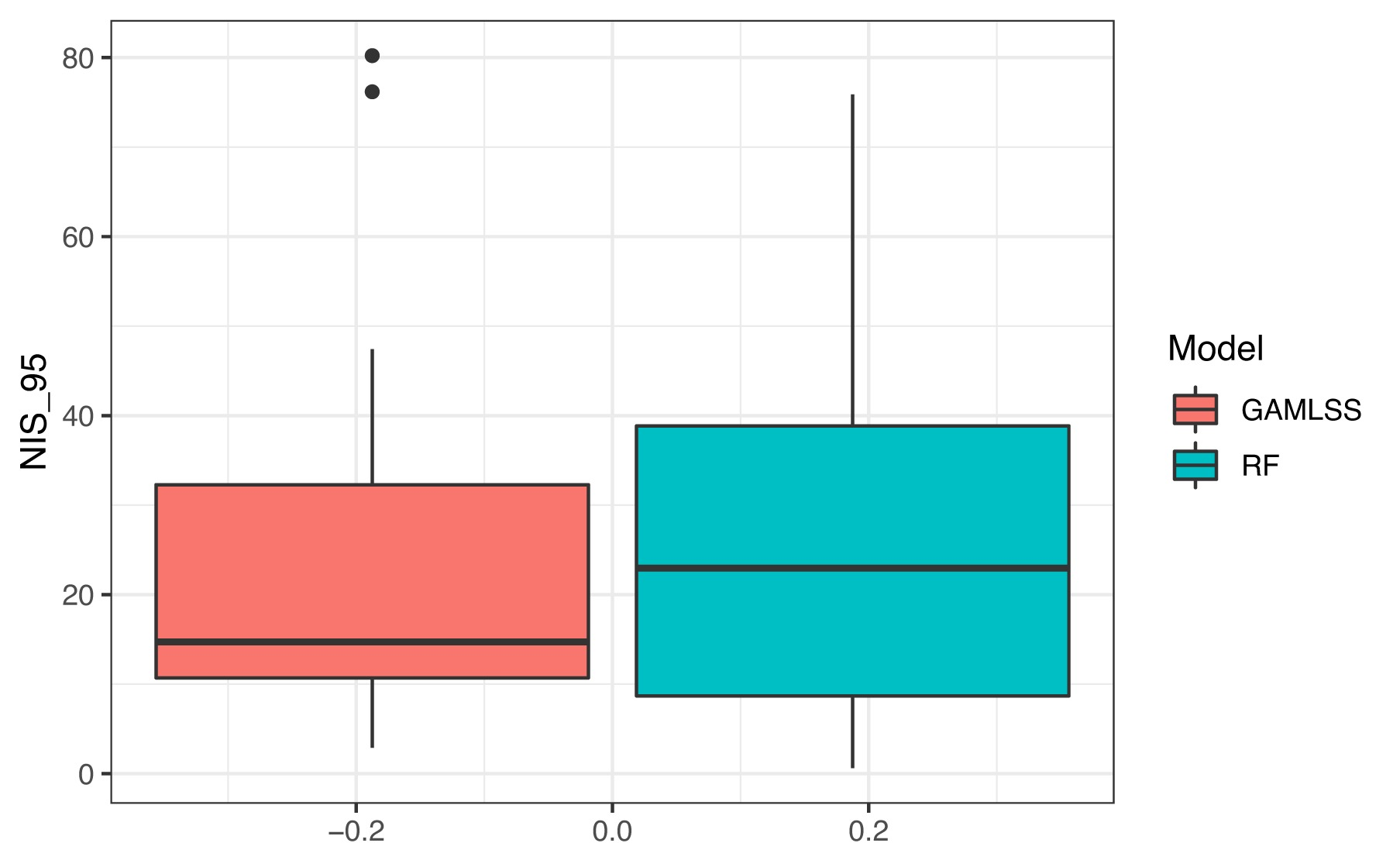

Supplement: S1 Fig — (TIF) [file pntd.0011047.s001.tif]

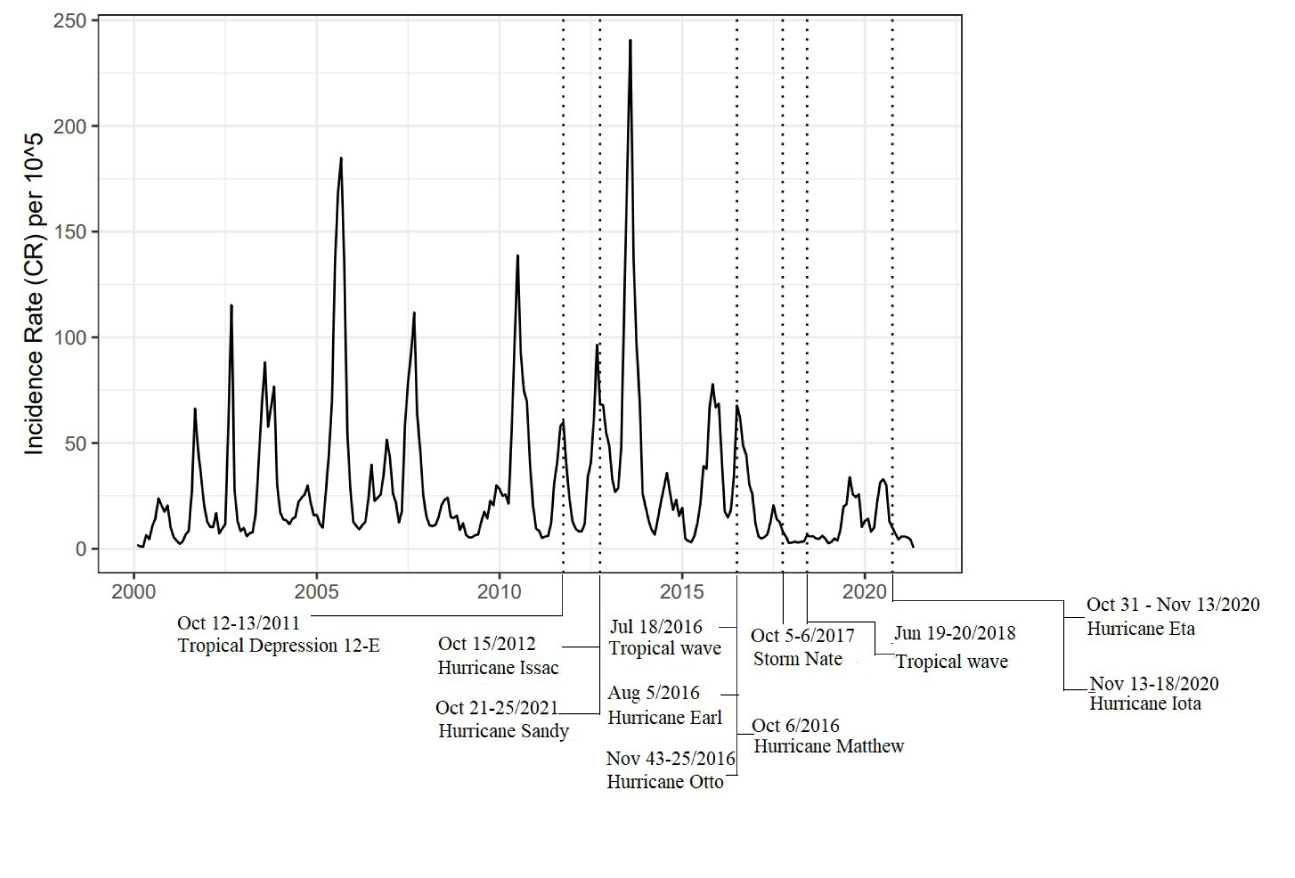

Supplement: S2 Fig — (TIF) [file pntd.0011047.s002.tif]

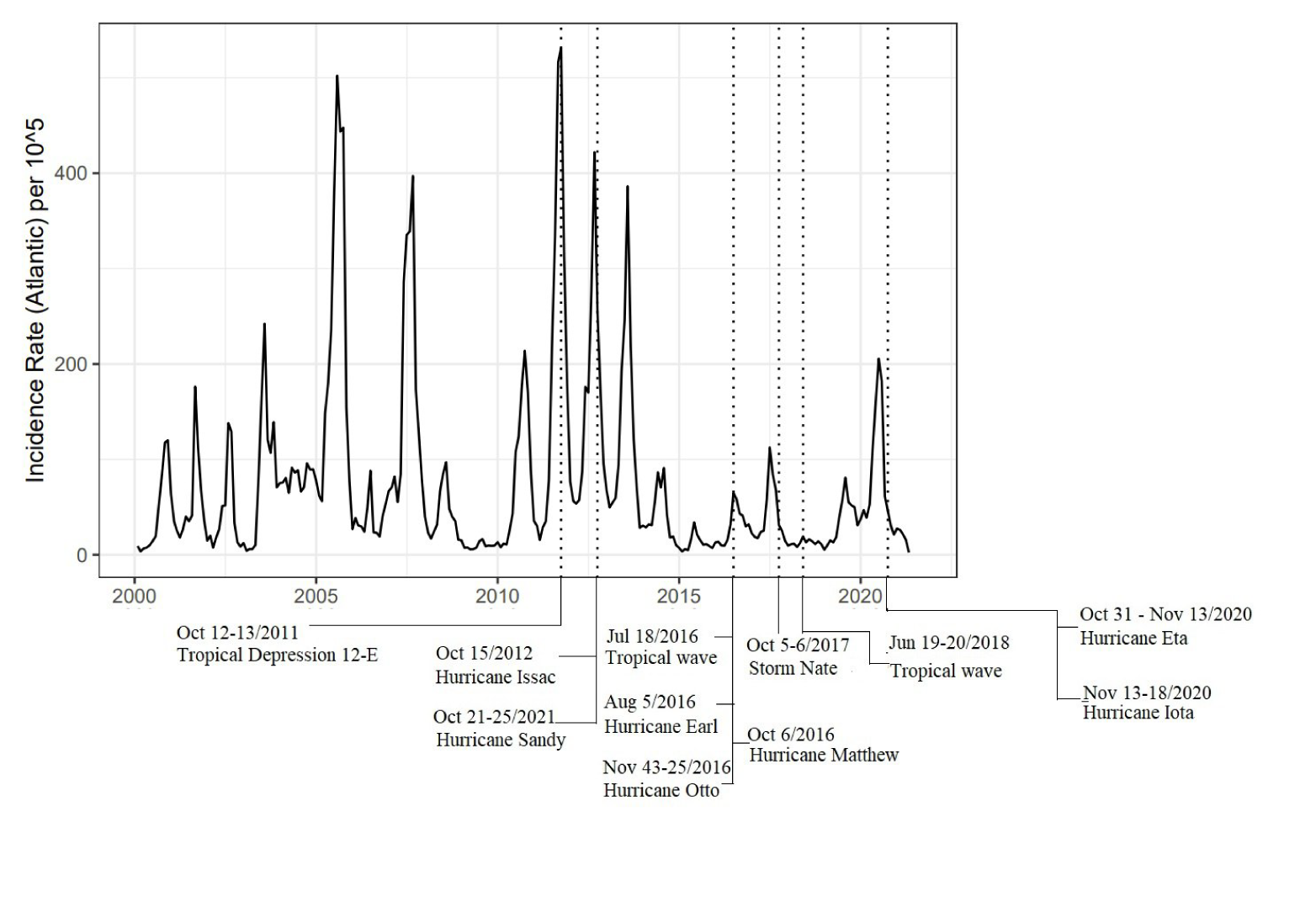

Supplement: S3 Fig — (TIF) [file pntd.0011047.s003.tif]

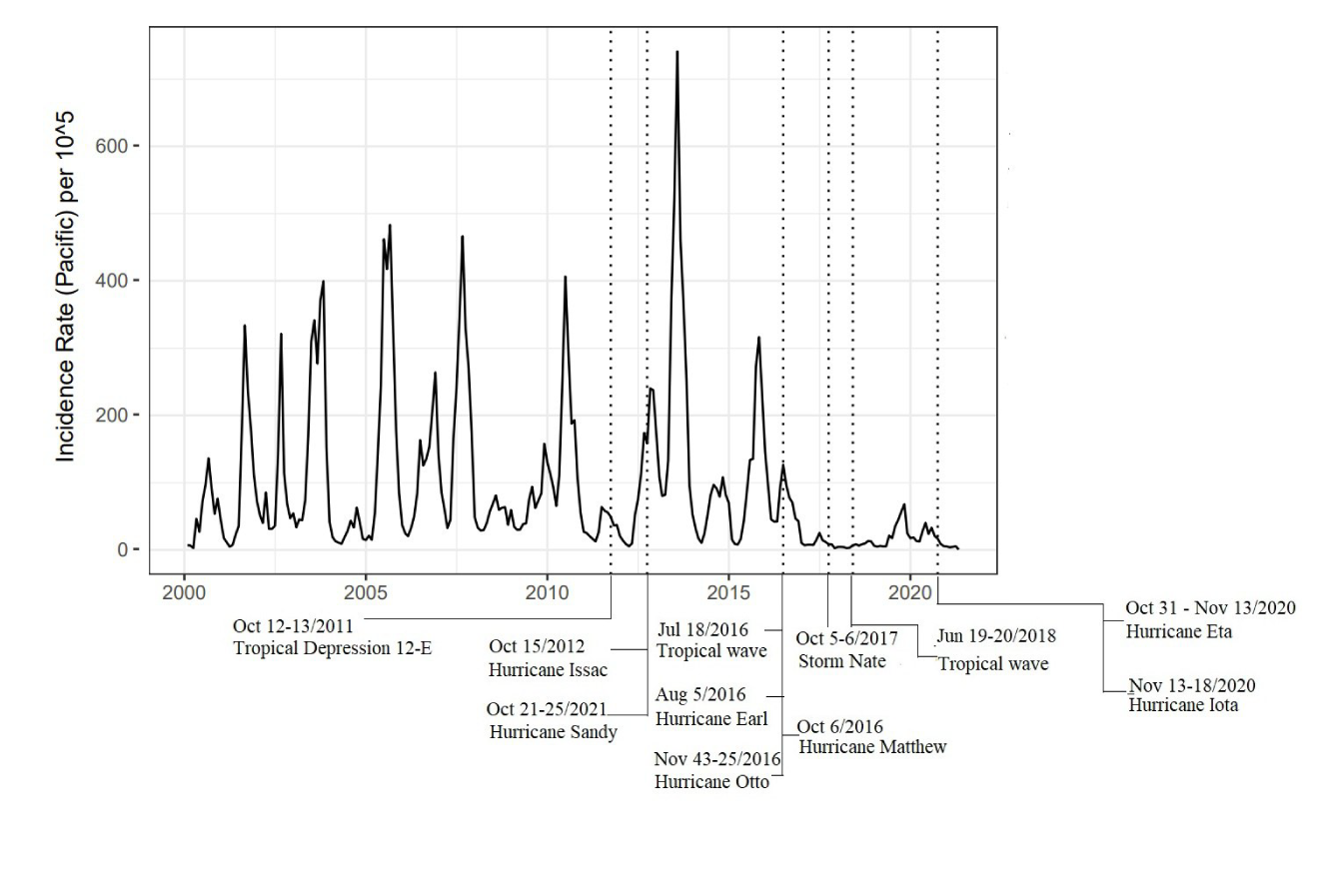

Supplement: S4 Fig — (TIF) [file pntd.0011047.s004.tif]
